# Adverse effects following COVID-19 vaccination in Iran

**DOI:** 10.1186/s12879-022-07411-5

**Published:** 2022-05-18

**Authors:** Ebrahim Babaee, Ali Amirkafi, Arash Tehrani-Banihashemi, Neda SoleimanvandiAzar, Babak Eshrati, Zahra Rampisheh, Mehran Asadi-Aliabadi, Marzieh Nojomi

**Affiliations:** 1 Preventive Medicine and Public Health Research Center, Psychosocial Health Research Institute, Department of Community and Family Medicine, School of Medicine, Iran University of Medical Sciences, Shahid Hemmat Highway, P.O Box: 14665-354, Tehran, 1449614535 Iran; 2 Present Address: Mazandaran University of medical Science, Sari, Iran, Mazandaran University of medical Science, Sari, Iran, Mazandaran Sari, Iran

**Keywords:** Adverse effect, Sputnik V, Sinopharm, AstraZeneca, Vaccine, COVID-19, Iran

## Abstract

**Background:**

Vaccination is a key intervention to prevent COVID-19. Many vaccines are administered globally, yet there is not much evidence regarding their safety and adverse effects. Iran also faces this challenge, especially as data regarding the Sputnik V vaccine is sparse. Therefore, the aim of this study is to determine the adverse effects of the most commonly used vaccines in Iran.

**Methods:**

Using a retrospective cohort study design, 6600 subjects aged 18 years or older who had received two doses of any of the three COVID-19 vaccines (Sinopharm, AstraZeneca, and Sputnik V) were selected using a random sampling method between March and August 2021. Subjects were asked about any adverse effects of the vaccines by trained interviewers via telephone interview. Vaccine-related adverse effects in individuals during the first 72 h and subsequently following both doses of the vaccines were determined. The demographic variables, type of administered vaccine, adverse effects, and history of the previous infection with COVID-19 were collected. Descriptive statistics (mean, standard deviation) and analytical statistics (Chi-squared and Wilcoxon tests) were performed at a 95% significance level using STATA software version 15 (STATA Corp, College Station, TX, USA).

**Results:**

From 6600 participants, 4775 responded (response rate = 72.3%). Of the participants, 1460 (30.6%) received the AstraZeneca vaccine, 1564 (32.8%) received the Sinopharm vaccine and 1751 (36.7%) received the Sputnik V vaccine. 2653 participants (55.56%) reported adverse effects after the first dose and 1704 (35.7%) after the second dose. Sputnik V caused the most adverse effects with 1449 (82.7%) vaccine recipients reporting symptoms after the first or second dose, compared with 1030 (70.5%) for AstraZeneca and only 585 (37.4%) for the Sinopharm vaccine. The most common adverse effects after the first dose were fatigue (28.37%), chill/fever (26.86%), and skeletal pain (22.38%). These three adverse effects were the same for the second dose, although their prevalence was lower.

**Conclusions:**

In this study, we demonstrate that the Sputnik V vaccine has the highest rate of adverse effects, followed by the AstraZeneca and Sinopharm vaccines. COVID-19 vaccines used in Iran are safe and there were no reports of serious adverse effects.

**Supplementary Information:**

The online version contains supplementary material available at 10.1186/s12879-022-07411-5.

## Background

Following the worldwide spread of COVID-19 and the confirmation of two deaths in Iran due to COVID-19, the epidemic in Iran was announced by the Ministry of Health on February 19, 2020 and the infection spread rapidly in 31 provinces after 15 days [1]. As of December 6, 2021, 6,134,465 cases and 130,200 deaths of COVID-19 have been confirmed [2].

As in most parts of the world, restrictions were imposed to control the spread of COVID-19 in Iran including restrictions on vehicle traffic in terms of time and geographical area based on the level of risk announced by the Ministry of Health, limiting the number of office staff at work, introducing teleworking, closure of jobs based on risk level by the Ministry of Health, mandatory use of masks in public places, and community education through mass media and health workers [3]. Each period of implanting these restrictions was followed by reduction in the number of cases and deaths; on the other hand, ignoring restrictions and neglecting COVID-19 prevention triangle (using a mask, social distancing, hands disinfection) lead to a new wave [4]. Although many therapeutic drugs have been proposed for COVID-19, more studies are still needed to determine their effectiveness and potency [5–7]; as a result, vaccine production and administration may be the best way to control the disease [8].

Therefore, one of the best strategies to control COVID-19 around the world could be public vaccination, a policy that could save millions of lives each year [9]. Currently, the best option to combat the spread of COVID-19 is using an effective and safe vaccine [10]. The adverse effects of vaccines should be identified and communicated transparently to maintain public’s trust in the vaccination programs.

Several companies around the world have developed COVID-19 vaccines. For example, the Sputnik V vaccine is an adenovirus vaccine and has 91.6% efficacy based on third phase clinical trial conducted in Russia [9]; it is currently used in 68 countries and the most common adverse effects of this vaccine are fatigue, joint pain, headache, muscle pain, chills, fever, nausea, and vomiting [10]. The BBIBP-CorV vaccine (Sinopharm), produced in China, is also an inactivated vaccine and shows 79.34% efficacy. In this vaccine, inactivated viruses retain the ability to reproduce in vivo with mild or asymptomatic symptoms. Reported adverse effects include pain around the injection site, fatigue, fever, headache, and lethargy [11].

To achieve the highest levels of immunity, the coverage and vaccination rate in the population is very important; in addition, there are only a few studies assessing COVID-19 vaccine side effects in Iran; which investigated health care workers [12, 13]. In the current study we aimed to assess the adverse effects of the vaccines in persons vaccinated with Sinopharm, AstraZeneca, and Sputnik V vaccines among general population. The results of this study will provide information to health policy makers on which kinds of vaccines could be used to ensure fewer adverse effects.

## Methods

### Design

Using a retrospective cohort study design, about 6600 subjects aged 18 years or older were included who had received two doses of COVID-19 vaccines. Vaccine recipients were enrolled for assessing the adverse effects. Data collection was carried out between March and August 2021.

### Data sources and subjects

COVID-19 vaccination in Iran was initiated on March 03, 2021, and this study was conducted on August 24, 2022. Subjects were selected by a random sampling method from the districts situated in west, north west and south west of Tehran (The Capital of Iran), in addition to three other towns in south west of Tehran; Iran University of Medical Sciences provide health services to the mentioned locations. All vaccinated adults aged 18 years and older were eligible for inclusion to the study. Subjects were vaccinated by trained health staff at the hospitals or other governmental health care services. This study was approved by The Research Ethics Committee of the Iran University of Medical Sciences (Ethical code: IR.IUMS.REC.1400.358) and was conducted based on the Helsinki Declaration.

Data was collected from the electronic national vaccination registration system. This system was established at the start of the vaccination program for COVID-19 to monitor uptake of second doses. The database includes other electronic health records of those receiving vaccinations and is updated when a person receives health services, such as vaccination. This information is accessible at the level of the University of Medical Sciences which are involved in both medical education and health services and also Ministry of Health level. We used the data from Iran University of Medical Sciences (IUMS) branch of this system, which includes the vaccination data for the population of the northwest and west of Tehran. Between the start of the vaccination program for COVID-19 and the beginning of the study, there were 1,863,101 sets of registration data in the IUMS branch of the national system. Using a simple random selection method, 6600 subjects were selected to assess the adverse events.

### Measurements and variables

We used a checklist to gather the data. Demographic variables, type of administered vaccine, use of sedative (in our questionnaire, sedatives were defined as the drugs to stop pain and/or fever, including NSAIDs, Paracetamol or injecting pain killers), occurrence of selected adverse effects, history of previous infection with COVID-19, and other reported variables were recorded. The guidelines of the studied vaccines were used to create a list of possible adverse effects. The main adverse effects reported were local reactions, and systematic reactions. In addition, severe neurological symptoms were questioned by asking “whether the participant had any severe neurological symptoms such as severe headache, blurred vision, diplopia, seizure or any similar symptoms”.

Subjects had received one of three types of vaccines: Sinopharm, AstraZeneca or Sputnik V. The first and second doses of Sinopharm and Sputnik V vaccines were administered at 4-week intervals and AstraZeneca at 3-month intervals. However, these time intervals were not strictly observed by some subjects who were found to have a longer interval between doses. Subjects were asked about the adverse effects of the vaccines by trained interviewers during a telephone interview. We obtained telephone numbers from the national register system. The interviewer first introduced themselves and asked the participants for informed consent. Once consent was given, the rate of vaccine adverse effects during both the first 72 h after receiving both doses of the vaccines, and subsequently was recorded. We excluded subjects with incomplete records from the study. These included people who either had incomplete information in the database or did not answer the phone.

### Statistical analysis

We used mean ± standard deviation (SD) and percentages to describe the quantitative and categorical variables respectively. The proportion of adverse effects was reported separately in each subgroup with related confidence intervals. The Chi-squared test was used to analyze the difference between qualitative variables; and Wilcoxon test was used, in addition to mean and median, to analyze and show the difference of paired quantitative variables which didn’t have a normal distribution. Hypothesis tests have been performed on a number of important variables that could affect the occurrence of adverse effects. All statistical analyses were performed at a 95% significance level using STATA software version 15 (STATA Corp, College Station, TX, USA).

## Results

In this study a total of 6600 COVID-19 vaccine recipients were enrolled for assessing the adverse effects. After two phone calls, 4775 vaccine recipients were interviewed and answered the self-reported questionnaire (response rate = 72.3%) to assess vaccine adverse effects after both first and second doses of vaccine. The mean age of study participants was 57.73 years (20–100 years). Fifty-five point forty six percent of participants were female, and 44.54% were male. Table 1 shows the demographic data of participants.

**Table 1:** Demographic characteristics of participants (n = 4775)

Variables					Groups		Number		Percent	
Age groups (years)					18–30		527		11.04	
					31–40		679		14.22	
					41–50		731		15.31	
					51–60		534		11.18	
					61–70		453		9.49	
					70<		1851		38.76	
Sex					Female		2648		55.46	
					Male		2127		44.54	
Vaccine					Sinopharm		1564		32.75	
					AstraZeneca		1460		30.58	
					Sputnik V		1751		36.67	
Job					Self Employed		98		2.05	
					Retired		1216		25.47	
					Unemployed		123		2.58	
					Nurse		386		8.08	
					General practitioner		217		4.54	
					Specialist Doctor		240		5.03	
					Housewife		958		20.06	
					Student		4		0.08	
					Health staff		480		10.05	
					Health expert		207		4.34	
					Administrative staff		846		17.72	
Education					Illiterate		535		11.20	
					High school		915		19.16	
					Diploma		852		17.84	
					University degree		2463		51.58	
					Missing data		10		0.21	
Underlying disease					Yes		1525		31.9	
					No		3250		68.1	
Previous COVID-19 infection					Yes		1118		23.41	
					No		3657		76.59	
Sensitive to substances					Nothing		4426		92.7	
					Drug		135		2.82	
					Food		202		4.23	
					Drug and Food		12		0.25	
Smoker					No		4327		90.61	
					Yes		444		9.29	
					Missing data		4		0.08	
Sedative use before and after vaccine					Before and after		171		3.58	
					Before		37		0.77	
					After		2163		45.29	
					No sedative		2402		50.30	
					Missing data		2		0.04	
Vaccinators informed participants about possible adverse effects					Yes		3877		81.19	
					No		898		18.81	
Sinopharm	18–30	31–40	41–50	51–60		61–70	> 70	Male		Female
Number	28	33	37	31		190	1245	761		803
Percent	0.02	0.02	0.02	0.02		0.12	0.80	0.49		0.51
AstraZeneca	18–30	31–40	41–50	51–60		61–70	> 70	Male		Female
Number	266	284	161	67		141	541	702		758
Percent	0.18	0.19	0.11	0.05		0.10	0.37	0.48		0.52
Sputnik V	18–30	31–40	41–50	51–60		61–70	> 70	Male		Female
Number	233	362	533	436		122	65	664		1087
Percent	0.13	0.21	0.30	0.25		0.07	0.04	0.38		0.62

According to the collected data, 3064 participants (64.2%) reported adverse effects in the first 72 h after the injection. Among the recipients, 2653 (55.56%) had adverse effects after the first dose and 1704 (35.7%) after the second dose. Table 2 shows the reported adverse effects. There were no reports of anaphylactic reactions, loss of consciousness, or internal bleeding or clot formation among the participants. In the 3064 participants with adverse effects, symptoms continued for more than 3 days in 210 (4.6%) people.

**Table 2:** Number of participants with specific adverse effects and respective percentage to the whole recipients

Variables	Sinopharm	AstraZeneca	Sputnik V	Total	P value
First 72 h, 1st dose					
Local reactions	55 (3.5%^a^)	107 (7.3%)	363 (20.7%)	525 (11%)	0.001
General fatigue	226 (14.5%)	353 (24.2%)	776 (44.3%)	1355 (28.37%)	0.001
Chills and fever	102 (6.5%)	657 (45%)	524 (29.9%)	1283 (26.86%)	0.001
Dizziness and headache	64 (4.1%)	235 (16.1%)	240 (13.7%)	539 (11.28%)	0.001
Skeletal pain	61 (3.9%)	405 (27.74%)	603 (34.4%)	1069 (22.38%)	0.001
Nausea	8 (0.51%)	51 (3.49%)	86 (4.91%)	145 (3.03%)	0.001
Diarrhea	2 (0.12%)	11 (0.75%)	35 (2%)	48 (1%)	0.001
Sleepiness	11 (0.7%)	17 (1.16%)	28 (1.6%)	56 (1.17%)	0.057
Loss of appetite	0	15 (1.02%)	11 (0.62%)	26 (0.054%)	0.001
Chest pain and dyspnea	1 (0.06%)	11 (0.75%)	6 (0.34%)	18 (0.37%)	0.008
Abdominal pain	0	6 (0.41%)	13 (0.74%)	19 (0.39%)	0.003
Severe neurological	0	3 (0.2%)	0	3 (0.06%)	0.033
No adverse effects	1137 (72.7%)	496 (34%)	489 (28%)	2122 (44.4%)	0.001
First 72 h, 2nd dose					
Local reactions	42 (2.7%)	50 (3.4%)	326 (18.6%)	418 (8.75%)	0.001
General fatigue	139 (8.9%)	219 (15%)	435 (24.8%)	793 (16.6%)	0.001
Chills and fever	40 (2.6%)	109 (7.5%)	387 (22.1%)	536 (11.22%)	0.001
Dizziness and headache	40 (2.6%)	78 (5.3%)	126 (7.2%)	244 (5.1%)	0.001
Skeletal pain	15 (1%)	128 (8.8%)	439 (25.1%)	582 (12.18%)	0.001
Nausea	2 (0.12%)	16 (1.1%)	33 (1.88%)	51 (1.06%)	0.001
Diarrhea	1 (0.06%)	7 (0.47%)	8 (0.45%)	16 (0.33%)	0.077
Sleepiness	10 (0.63%)	21 (1.43%)	18 (1.02%)	49 (1.02%)	0.093
Loss of appetite	0	1 (0.06%)	3 (0.17%)	4 (0.08%)	0.228
Chest pain and dyspnea	0	1 (0.06%)	2 (0.11%)	3 (0.06%)	0.422
Abdominal pain	0	1 (0.06%)	13 (0.74%)	14 (0.3%)	0.001
Severe neurological	0	0	2 (0.11%)	2 (0.04%)	0.178
No adverse effects	1295 (82.8%)	1057 (72.4%)	719 (41.06%)	3071 (64.31%)	0.001

^a^As each person could have more than one adverse effect, the percentage is shown separately for each adverse effect; which is calculated through dividing the number of a particular adverse effect by the number of participants receiving a particular vaccine; not by the total number of adverse effects

Twenty-three point four percent of the participants had a history of COVID-19 infection, either clinically diagnosed or PCR confirmed. Occurrence of adverse effects was significantly higher in participants with a history of COVID-19 infection who received the Sputnik V or AstraZeneca vaccine than those who reported no history of infection (P = 0.001); on the other hand, there was no difference among Sinopharm recipients (P = 0.33). People who used sedatives after vaccine injection reported adverse effects more frequently than the people who did not use them; this finding was observed with all three vaccines (P = 0.001).

### Sinopharm

Participants who received the Sinopharm vaccine were older (mean = 73.54 years) and 860 (54.9%) had underlying diseases, which more than the users of other two vaccines. The most prevalent underlying disease was diabetes (355 cases).

Nine hundred and seventy nine (62.6%) of the Sinopharm recipients didn’t report any adverse effects after neither of the doses. The most frequent adverse effects after both the first and second doses of Sinopharm vaccine were fatigue (1st dose: 14.5%, 2nd dose: 8.9%), chill/fever (1st dose: 6.5%, 2nd dose: 2.6%), dizziness/headache (1st dose: 4.1%, 2nd dose: 2.6%) and local reactions (1st dose: 3.5%, 2nd dose: 2.7%) (Table 2).

The number of adverse effects per participant, after the first dose of vaccine (mean = 0.38, median = 0, max = 5) was significantly higher than the second dose (mean = 0.21, median = 0, max = 4) (P = 0.001). Among 30–50 year old participants, chill/fever was the most frequently reported symptom (12.8%), whereas among participants aged 50–60 years, dizziness/headache (16.1%), and skeletal pain (16.1%) were the most prevalent symptoms. Fatigue is the most common in other age groups (25% in 18–30 years, 16.8% in 60–70 years and 14.2% in older than 70 years group). The full subgroup data during and after 72 h of the first and second dose are available in Additional file 1.

### Sputnik V

Table 2 presents most common adverse effects of the first dose of the Sputnik V vaccine in participants. Three hundred and three individuals (17.3%) of the Sputnik V vaccine recipients did not have any adverse effects after both doses.

Fatigue (1st dose: 44.3%, 2nd dose: 24.8%), local reactions (1st dose: 20.7%, 2nd dose: 18.6%), and chill/fever (1st dose: 29.9%, 2nd dose: 22.1%), were the most common adverse effects after the first and second dose of the vaccine. Younger recipients reported more adverse effects than the older age groups (86.4% in participants younger than 50 vs 76% in younger ones).

Female participants reported more adverse effects per participant compared to males (87.1% vs 75.6%, P = 0.001). Occurrence of adverse effects per participant was higher (P = 0.001) after the first dose (mean = 1.69, median = 2, max = 10) compared to the second dose (mean = 1.1, median = 1, max = 6). In contrast to other two vaccines, Sputnik V recipients who smoked reported fewer adverse effects (19.2% compared to 28.7%).

### AstraZeneca

Of the AstraZeneca vaccine recipients, 430 (29.4%) didn’t have any adverse effects after vaccination. The most prevalent adverse effects for both doses were chill/fever (1st dose: 45%, 2nd dose: 7.5%), skeletal pain (1st dose: 27.7%, 2nd dose: 8.8%) and fatigue (1st dose: 24.2%, 2nd dose: 15%).

The majority of recipients were older than 70 years of age (37%), and the percentage of symptoms was lower in older recipients compared to younger age groups (55.3% in participants older than 60 years old vs 83.9% in younger ones, P value 0.001). Also, more symptoms occurred in the female population compared to the male group (77% vs 63%, P value = 0.001).

Comparing the number of adverse effects after the first and second dose of AstraZeneca vaccine using the Wilcoxon test showed that first dose cause more adverse effects per participant (mean = 1.45, median = 1, max = 8) than the second dose (mean = 0.48, median = 0, max = 6) (P = 0.001).

## Discussion

In this cross-sectional study, we investigated the adverse effects of the most commonly used COVID-19 vaccines in Iran; Sinopharm, AstraZeneca, and Sputnik V vaccines. The study included 4775 participants who had received both doses of the vaccine. During the 72 h after the first dose, the most prevalent symptoms were general fatigue (1355 cases, 28.3%), chill and fever (1283 cases, 26.8%), and skeletal pain (1069 cases, 22.3%). During the first 72 h after the second dose, the top three symptoms were the same. However, the order and prevalence are different; 793 (16.6%) had general fatigue, 582 (12.2%) had skeletal pain and 536 (11.2%) had chill and fever. After the first 72 h following injection, the total number of symptoms was much lower; 157 participants for the first dose and 42 after the second one.

Sputnik V was the first COVID-19 vaccine in the world to be registered [14]. However, few studies have been conducted to assess the efficacy and safety of the Russian vaccine. Most of the available data is through phases I, II and III of the clinical trials [11]. Main studies which assessed this vaccine’s adverse effects were conducted by Pagotto et al. in Argentina and by Babamahmoodi et al. and Zare et al. in Iran; all investigating health care workers [12, 13, 15].

We found that the overall percentage of participants having adverse effects was 82.7%, which is higher than 64.7% reported in the phase III trial. Also in comparison to post authorization studies, it is very close to Zare’s study which reported 81.9% of participants having symptoms, whereas Pagotto reported this number to be 71.3% [13, 15]. Babamahmoodi did not mention the overall incidence; however female and male comparison is conducted, where findings are similar to our study; women were reported to have more adverse effects following the first dose, but men had more symptoms after the second dose. The most common symptoms included fatigue, skeletal/muscle pains, chill/fever, injection site reactions, and headache; which were similar to previous studies, yet the order is different [12].

Sinopharm is the most commonly administered vaccine in Iran [16]. We found that for both doses, females reported more adverse effects than males; which is similar to the results of a study conducted in United Arab Emirates [17]. Fatigue, chill/fever, headache, and injection site reactions are the most frequently reported adverse effects. These adverse effects are similar to those found in Indian and Iraqi studies [16, 18]; but the rate of these effects in our study is lower and a large proportion of participants did not report any symptoms for either the first (72.6%) or second dose (82.8%). Furthermore, in contrast to the study conducted by Almufty et al. in Iraq [16], having positive history of COVID-19 infection reduced the rate of adverse effects, although the difference is only 3.5%. In comparison to the clinical trial phase 1/2, overall percentage of participants with adverse effect is close to current study (39% vs 37.4%). However according to the trial, older participants had less adverse effects than the young ones after both doses; whereas in our study for the first dose, adverse effects occurrence decreased with age; and for the second dose increased with age [19].

AstraZeneca vaccine is the second most frequently administered in Iran. Chill/fever, skeletal pain, fatigue, and headache were the most common adverse effects respectively and were present in both males and females for either the first and second dose. In a prospective observational study in the United Kingdom, local reactions were reported to be the most prevalent adverse effect, followed by headache and fatigue [20]. A prominent finding of our study was the increased number of adverse effects in participants with previous comorbidities, which is in line with Iraqi study [16]. This effect is highlighted for the second dose where there is a 73% increase in adverse effects for those with a previous comorbidity. One of the main concerns was the reports of thrombotic adverse effect after vaccine injection [21]. However, in the current study, there were no serious adverse effect related to thrombosis.

Overall, the participants vaccinated with Sputnik V reported a higher rate of symptoms, followed by those who received the AstraZeneca vaccine. The Sinopharm vaccine had the lowest number of reported adverse effects. This is similar to other studies for Sinopharm and AstraZeneca vaccines [22, 23]. Yet in contrast to our study, Zare found that Sputnik V vaccine caused less adverse effects than AstraZeneca [13]. We also found that second doses of Sinopharm and AstraZeneca vaccines produce fewer adverse effects. However, the Sputnik V vaccine is the opposite that the second dose caused more adverse reactions. Which are in line with other similar studies [11, 24]. Sedatives and paracetamol, are considered a possible way to reduce adverse effects [25], yet in our study number of adverse effects was higher in people who used sedatives or paracetamol. Since the exact time of usage was not recorded, it is possible that participants started using them after the onset of symptoms.

As this is one of the first studies conducted in Iran to assess the adverse effects of the most frequently administered COVID-19 vaccines, we encountered some limitations for designing and implementing the study. According to the study design, there weren’t any control groups. A major limitation of this study was not assessing the severity or seriousness of the adverse effects. Another limitation regarding the sample was selecting them only from Tehran; despite being the capital and including people from various parts of Iran, their proportion is not a proper representative of the whole Iranian population; however we tried to select from various demographic groups. Also, due to the short interval between start of mass vaccination and the time when study was conducted, only the common and short-term adverse effects are assessed. To investigate possible mid- and long-term effects, a longer follow-up period is required [26]. Therefore, in the second phase of this study and according to WHO guideline for safety signal detection of COVID-19 vaccines [27], a case–control method could be used to investigate more serious, rare, and long-term reactions of vaccines. According to the sample size, it was not possible to study very rare adverse effects; for doing so, a larger sample is needed. In addition 25% of participants did not answer whether due to not answering their phones or being unwilling to participate; follow up calls with a clearer explanation about the logics behind this study could increase the response rate. Finally, in this study, because the mean age of participants was significantly different between the three vaccines groups, this issue could affect the results of the study.

## Conclusion

All three vaccines proved to be safe and most of the adverse effects were mild and resolved within the first 72 h following vaccine administration. Overall, local reactions, fatigue, chill, fever, muscular and skeletal pain, headaches, and dizziness were the most prevalent ones. Sputnik V caused the most symptoms, followed by the AstraZeneca and Sinopharm vaccines. However, adverse effects differed for each vaccine and each dose according to sex, age group, previous COVID-19 infection, and use of sedatives or paracetamol. To continue the path towards more evidence-based decision making, further studies should be carried out to assess rare, long-term and severe adverse effects. It is essential to conduct such studies to tackle attitudes towards vaccine hesitancy and increase vaccine coverage.

## Supplementary Information

**Additional file 1.** Proportion and confidence interval of most common side effects during the first 72 h after the injection of the first dose in demographic subgroups.

## Data Availability

Data is available via contact with Dr. Babak Eshrati who is one of the colleagues of the study and also one of the co-authors. His email is: Eshrati.b@iums.ac.ir.

## Abbreviations

IUMS: Iran University of Medical Sciences
SD: Standard deviation
WHO: World Health Organization
TX: Texas
USA: United States of America
